# SAP97 rs3915512 Polymorphism Affects the Neurocognition of Schizophrenic Patients: A Genetic Neuroimaging Study

**DOI:** 10.3389/fgene.2020.572414

**Published:** 2020-10-08

**Authors:** Xusan Xu, Bin He, Zhixiong Lin, Xiaoxia Wang, Jingwen Yin, Xudong Luo, Shucun Luo, Chunmei Liang, Xia Wen, Susu Xiong, Dongjian Zhu, Jiawu Fu, Dong Lv, Zhun Dai, Juda Lin, You Li, Wubiao Chen, Zebin Luo, Yajun Wang, Guoda Ma

**Affiliations:** ^1^Institute of Neurology, Affiliated Hospital of Guangdong Medical University, Zhanjiang, China; ^2^Maternal and Children’s Health Research Institute, Shunde Women and Children’s Hospital, Guangdong Medical University, Foshan, China; ^3^Department of Radiology, Affiliated Hospital of Guangdong Medical University, Zhanjiang, China; ^4^Department of Psychiatry, Affiliated Hospital of Guangdong Medical University, Zhanjiang, China; ^5^Clinical Research Center, Affiliated Hospital of Guangdong Medical University, Zhanjiang, China

**Keywords:** schizophrenia, SAP97, rs3915512, cognitive functions, ALFF, RSFC

## Abstract

Our previous study suggested that the synapse-associated protein 97 (SAP97) gene rs3915512 polymorphism may influence neurocognition in schizophrenia patients. Neuroimaging studies have shown a possible association between cognitive function and brain activity/connectivity. Considering the poor understanding of whether the disease state and SAP97 rs3915512 polymorphism have interactive effects on brain activity/connectivity, 52 first-episode schizophrenia (FES) patients and 52 healthy controls were genotyped using blood DNA samples and underwent magnetic resonance imaging scanning. A two-way ANCOVA model was performed with rs3915512 genotypes and disease state as the between-subject factors. A significant disease × SAP97 interactive effect was found for the amplitude of low-frequency fluctuation (ALFF) in the right supplementary motor area, left rolandic opercularis area (ROC-L), and bilateral middle occipital gyrus (MOG). In addition, among auditory/visual-related brain areas, a significant interactive effect was found for resting-state functional connectivity (RSFC) between the MOG-L and bilateral superior temporal gyrus (STG) in the STG-L with ROC-R, right cuneus (Cu-R), left fusiform (Fu-L), and left lingual gyrus (LG-L). Positive correlations were found between ALFF in the ROC-L and motor speed scores, between RSFC in the STG-L and LG-L and between Brief Assessment of Cognition in Schizophrenia verbal memory scores in FES. The SAP97 rs3915512 polymorphism may affect neurocognitive function in patients with schizophrenia by changing the brain activity and connectivity of auditory/visual-related brain areas.

## Introduction

Cognitive impairments mainly affect memory, attention, information integration, and execution (Harvey et al., 2001). Cognitive impairments occur in up to 85% of patients with schizophrenia and have been described as a core manifestation of schizophrenia (Mallet et al., 2020).

The synapse-associated protein 97 (SAP97) gene, located at the schizophrenia susceptibility locus 3q29, was found to have reduced mRNA (Uezato et al., 2015) and protein expression in patients with schizophrenia (Toyooka et al., 2002). As a scaffold protein highly enriched in postsynaptic density, SAP97 can interact with many neurotransmitter receptors [i.e., the *N*-methyl-D-aspartate receptor (NMDAR; Sato et al., 2008), α-amino-3-hydroxy-5-methyl-4-isoxazole propionic acid receptor (AMPAR; Waites et al., 2009), and serotonin receptor (5-HTR; Dunn et al., 2014)]. In addition, dopamine receptor D4 can activate SAP97 through calcium/calmodulin kinase II (CaMKII) in the low activity of prefrontal neurons (Yuen and Yan, 2011). SAP97 can also bind to a variety of potassium channels that have been shown to participate in the regulation of cognition function (Grube et al., 2011). SAP97 may change the cognition of schizophrenic patients by interacting with neurotransmitter receptors and potassium channels. Our previous study showed that schizophrenic patients with the SAP97 rs3915512 TT genotype have higher cognitive function scores than those carrying the A allele (Xu et al., 2018).

Numerous neuroimaging studies have highlighted various abnormal regional activities and widespread dysconnectivity in schizophrenic patients contributing to heterogeneous and cognitive dysfunction (Cheng et al., 2015). Uezato et al. (2015) found that the T > A variation of rs3915512 may truncate the SAP97 protein. Therefore, we speculate that the single nucleotide polymorphism (SNP) may influence the function of SAP97, which may contribute to the abnormal distribution of neurotransmitter receptors, disturbance of neuroelectric activity and functional disconnections. These results prompted us to analyze the role of SAP97 genetic variations in the brain activity/connectivity of patients with schizophrenia. Considering that the results may be influenced by antipsychotic therapy administration, we recruited FES patients in the present study.

## Materials and Methods

### Subjects

This study was approved by the Ethics Committee of the Affiliated Hospital of Guangdong Medical University. All participants or their families gave written informed consent.

The 104 subjects involved in this study came from our previous cohort study (Xu et al., 2018). All subjects were recruited from the Department of Psychiatry and the Health Examination Center of the Affiliated Hospital of Guangdong Medical University. These enrolled individuals were all unrelated southern Han Chinese and met the diagnostic criteria of schizophrenia as described previously (Yin et al., 2019). Demographic information on the subjects was collected, including gender, age, nationality, education, family history, course of the disease, and age of onset. Meanwhile, the neurocognitive function of the patients was evaluated by the Brief Assessment of Cognition in Schizophrenia (BACS) scale (Keefe et al., 2004).

### Genotyping

Genomic DNA was extracted according to the instructions provided by the whole blood DNA extraction kit (Tiangen Biotech, Beijing, China). The 104 individuals were genotyped for rs3915512 using the improved multiplex ligation detection reaction (imLDR) technique (Genesky Biotech, Shanghai, China) described in our previous study (Xu et al., 2020).

### Imaging Data Acquisition

Images were acquired in a 3.0 T GE Discovery MR750 scanner (GE Healthcare Systems, Milwaukee, WI, United States) system with an 8-channel head coil, and the scanner was located at the Radiology Department of the Affiliated Hospital of Guangdong Medical University. All subjects were asked to keep their eyes closed, relax, stay awake, and minimize mental activity during the resting state. Resting-state functional magnetic resonance imaging (fMRI) was used to assess brain region activity based on the principle of blood oxygenation-level-dependent (BOLD) contrast enhancement (Fryer et al., 2015). Resting-state functional connectivity (RSFC) was assessed by the time dependence of low-frequency (0.01–0.10 Hz) oscillations of BOLD signals between different brain regions (Yu et al., 2012).

Blood oxygenation-level-dependent-fMRI data acquisition was performed using an echo planar imaging (EPI) sequence with the following parameters: time of echo (TE) = 30 ms, time of repetition (TR) = 2000 ms, flip angle (FA): 90°, scanning slice: 38 slice, slice thickness: 3.6 mm, slice interval: 0.6 mm, field of view (FOV) = 230 mm × 230 mm, matrix: 64 × 64, scanning time: 8 min, 240 dynamics.

T1-weighted data acquisition was performed using a three-dimensional (3D) fast field echo (FFE) pulse sequence with the following parameters: TE = 3.18 ms, TR = 8.16 ms, FA: 90°, scanning slice: 172 slices, slice thickness: 1 mm, slice interval: 0 mm, FOV = 512 mm × 512 mm, matrix: 256 × 256.

### Data Preprocessing

Imaging data processing was performed by Statistical Parametric Mapping 12 (SPM12, Wellcome Trust Centre of the University College London), RESTplus 1.2 and data processing assistant for resting-state fMRI (DPARSF_V2.3, Cognitive and Brain Diseases Centre of the Hangzhou Normal University), which was implemented on MATLAB 2012a. Major steps for data preprocessing include (1) converting DICOM Format Functional Data into NIfTI Format Data; (2) removing the data of the first 10 time points for each scan; (3) slice timing correction; (4) rigid-body head motion correction (2.5 mm displacements and 2.5° rotations); (5) normalizing to the Montreal Neurological Institute (MNI) template space, resampled with voxels of 3 mm × 3 mm × 3 mm; (6) spatial smoothing [6 mm full width at half maximum (FWHM) Gaussian kernel]; (7) filtering (0.01–0.08 Hz); and (8) nuisance signal regression including head motion parameters were calculated using the Friston 24 model, global signal, white matter signal, and cerebrospinal fluid signal.

Amplitude of low-frequency fluctuation values were obtained using the RESTplus 1.2 toolkit. Seed-based region of interest (ROI) wise functional connectivity analysis was performed by placing seeds with the 22 MNI coordinates in the auditory-related (bilateral rolandic opercularis area, supramarginal gyrus, Heschl gyrus, and superior temporal gyrus) and visual-related (bilateral calcarine, cuneus (Cu), lingual gyrus, superior occipital gyrus, middle occipital gyrus, inferior occipital gyrus, and fusiform gyrus) brain areas defined by previous researchers (specific MNI coordinates are shown in Supplementary Table 1) (Power et al., 2011). The time process signals around these coordinates with a radius of 6 mm were extracted, and the connections within these 22 functional nodes were analyzed. For the individual seed connectivity map, Fisher’s r-to-z transformation was used.

### Statistical Analyses

Because the AA genotype is very few, we merged the AA and TA genotypes into the A carrier group for analysis. Quantitative data are expressed as the means ± standard deviations (SD). The Hardy–Weinberg equilibrium (HWE), genotype and allele distributions were compared by Pearson’s Chi-square test. To test the effect of genotype on phenotypes, an independent sample *T* test was conducted with the genotype as the fixed factor, and age, age at onset, duration, years of education, and BACS scores (seven index scores) were the dependent factors. To obtain the MNI coordinates of interactive brain regions, the ALFF brain maps were analyzed by two-way factorial analyses of 2 × 2 ANCOVA [diagnosis (FES vs HC) × genotype (TT vs TA + AA)] and Monte Carlo multiple correction (Alphasim: rmm = 6, cluster size > 33). Then, the anatomical location of interactive brain regions in Anatomical Automatic Labeling (AAL) and Brodmann Area (BA) was located by the RESTplus 1.2 toolkit and was displayed by the Xjview 8.1 image viewing tool.^1^ With the ALFF value and RSFC value in each voxel as the independent variables, the interactive effect of genotype and disease was examined by 2 × 2 ANCOVA, with age, gender and educational year as covariates. To explore the details of the interactive effects, *post hoc* t-test analysis was used. All of the statistical analyses were performed by using SPSS 21.0 software, and the statistical significance was set at *P* < 0.05, Bonferroni correction except for obtaining the MNI coordinates of interactive brain regions.

### Correlation Analysis Between Brain Activity and Cognitive Scores

With age, gender and education as covariates, we used Spearman correlation to measure the relationship between the brain activity (ALFF value and RSFC value) and the BACS scores for each patient group. Statistical significance was defined as *P* < 0.05. To correct the results, Bonferroni correction was used.

## Results

Fifty-two FES patients (30 males and 22 females, mean age 27.29 ± 8.21 years, mean educational level 10.60 ± 2.70 years) and 52 HC patients (23 males and 29 females, mean age 29.17 ± 8.48 years, mean educational level 11.62 ± 2.61 years) were enrolled in this study. Gender, age, and education of the HC matched well with the FES (*P* = 0.252, 0.107, and *P* = 0.053, respectively). Genotype distributions and allele frequencies of rs3915512 showed no significant difference between the HC and FES groups (Table 1). Although the patients with the A allele in rs3915512 still had lower average BACS scores than those with the TT genotype, as in a previous study, no significant difference was found in these *P*-values (*P* > 0.05) (Table 2).

**TABLE 1:** Genotyping and allele distribution of SAP97 rs3915512 in HC and FES.

Group	HC	FES		*P* value
*n*	52	52		
HWE	0.484	0.324		
Age (years)	29.17 ± 8.48	27.29 ± 8.21	*F* = 0.73	0.252
Male/female	23/29	30/22	χ^2^ = 1.89	0.107
Education (years)	11.62 ± 2.61	10.60 ± 2.70	*F* = 0.50	0.053
TT	30	28		
TA/AA	19/3	22/2	χ^2^ = 0.16	0.693
MAF	0.24	0.25		
T	79	78		
A	25	26	χ^2^ = 0.03	0.872

*HC, healthy control; FES, first episode schizophrenia; HWE, Hardy–Weinberg Equilibrium; MAF, minor allele frequency.*

**TABLE 2:** Cognitive function of the schizophrenic patients and distribution by genotypes of the rs3915512 polymorphism in previous and present study.

	rs3915512					
	Previous study			Present study		
BACS	TT (*n* = 177)	TA + AA (*n* = 182)	*P* value	TT (*n* = 17)	TA + AA (*n* = 15)	*P* value
Working memory	17.04 ± 8.97	15.38 ± 8.87	0.079	23.47 ± 5.04	19.38 ± 6.28	0.067
Semantics fluency	31.16 ± 12.14	28.27 ± 12.39	**0.027**	33.77 ± 9.89	30.92 ± 6.16	0.393
Letter fluency	10.69 ± 5.97	9.20 ± 5.29	**0.013**	12.33 ± 5.23	12.08 ± 2.97	0.884
Verbal memory	26.46 ± 15.27	20.38 ± 12.49	**0.000**	37.27 ± 8.75	31.78 ± 8.79	0.180
Motor speed	50.68 ± 17.45	50.01 ± 17.00	0.712	45.35 ± 14.82	43.10 ± 14.06	0.701
Reasoning and problem solving	8.06 ± 6.10	7.44 ± 6.71	0.359	15.58 ± 5.18	12.64 ± 4.90	0.177
Attention and processing speed	23.25 ± 13.20	20.13 ± 13.38	**0.027**	33.46 ± 9.79	32.58 ± 9.37	0.821

*BACS, Brief Assessment of Cognition in Schizophrenia. The data of previous study comes from Xu et al. (2018). The bold values mean statistically significant (P < 0.05).*

**FIGURE 1:**
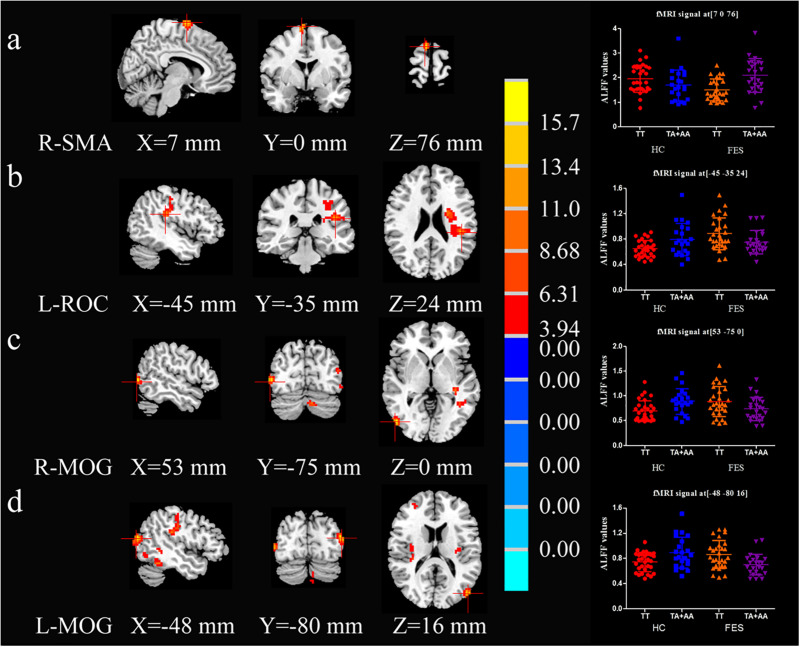
Brain maps of ALFF-differentiated regions with interactive effects. R-SMA **(A)**, L-ROC **(B)**, R-MOG **(C)**, and L-MOG **(D)** exhibited interactive effects between the SAP97 rs3915512 genotype and disease (2 × 2 ANCOVA *P* < 0.05, Alphasim correction, Cluster Size > 33). The red color indicates a significantly increased ALFF value in the brain area on the left. The number below the brain map is the MNI coordinate, the chromaticity bar of the *F* value is in the middle, and the signal distribution scatter map of the brain region with a significant interactive effect of the ALFF value is on the right.

As shown in Supplementary Table 2 and Figure 1, the interactive effect of ALFF values between diagnosis (HC and FES) and SAP97 genotype (TT and TA + AA) showed significant differences in the right supplementary motor area (SMA-R), left rolandic opercularis area (ROC-L), middle occipital gyrus (MOG)-R, and MOG-L (*P* = 3.30E-04, 0.001, 8.67E-04, and 1.40E-04, respectively, AlphaSim correction, Cluster Size > 33). Compared with the A allele, the patients with the TT genotype showed reduced ALFF values in the SMA-R (*P* = 4.52E-04) and higher ALFF values in the ROC-L, MOG-R, and MOG-L (*P* = 0.024, 0.036 and 0.004, respectively) in the *post hoc* analysis of genotype (Supplementary Table 2).

**FIGURE 2:**
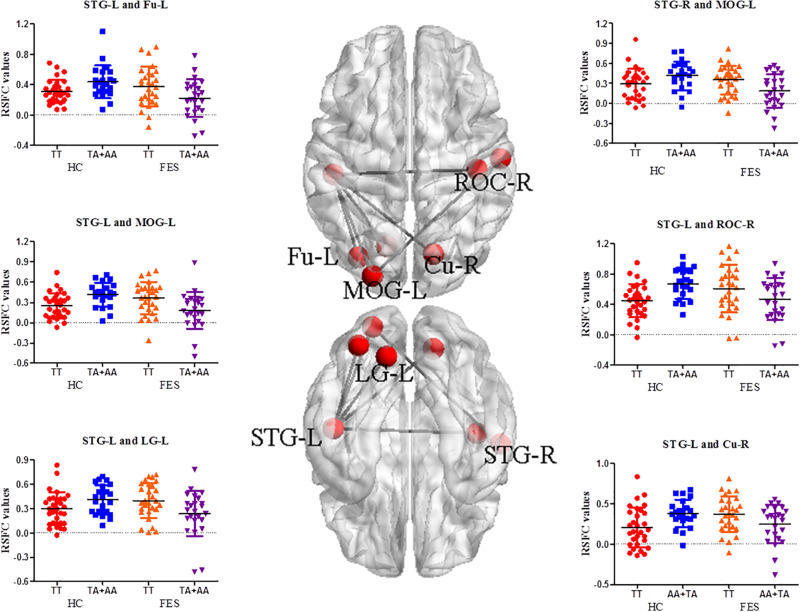
RSFC analysis between different functional network points in the auditory/visual-related brain areas in FES. The nodes on the top side and bottom side of the brain model in the middle were the functional brain areas with interactions, and the edges were the mean *Z* values between the two functional brain areas. The signal distribution scatter map of the functional connectivity with a significant interactive effect of the RSFC value is on the left and right (BrainNet Viewer software).

Significant interactive effects in RSFC of the MOG-L were observed in the bilateral superior temporal gyrus (STG) (*P* = 9.64E-04 and 9.08E-05, respectively), and RSFC of the STG-L was found in ROC-R, Cu-R, left fusiform (Fu-L), and left lingual gyrus (LG-L) (*P* = 2.77E-04, 6.46E-04, 0.001, and 1.85E-03, respectively) (Supplementary Table 3 and Figure 2). As shown in Supplementary Table 3, the TT genotype showed higher RSFC between the MOG-L and the bilateral STG (*P* = 0.006 and 0.003, respectively), STG-L and ROC-R (*P* = 0.031), STG-L and Cu-R (*P* = 0.031), STG-L and Fu-L (*P* = 0.009), and STG-L and LG-L (*P* = 0.007) in the *post hoc* analysis of genotype.

**FIGURE 3:**
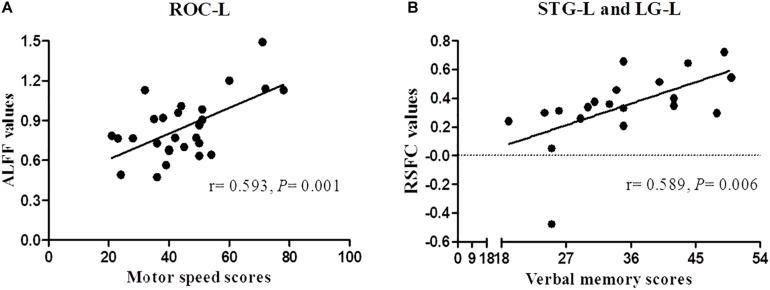
Correlation analysis between ALFF/RSFC and BACS scores of patients with schizophrenia. Panels **(A,B)** represent a significant positive correlation between ALFF/RSFC and BACS scores after Bonferroni correction (P < 0.007).

The Spearman correlation revealed a significant positive correlation between ALFF of ROC-L and BACS motor speed scores (*r* = 0.593, *P* = 0.001), MOG-R and BACS affective verbal memory scores (*r* = 0.468, *P* = 0.038), between RSFC of STG-L and Cu-R (r = 0.527, *P* = 0.017), STG-L and Fu-L (r = 0.469, *P* = 0.037), STG-L and LG-L (r = 0.589, *P* = 0.006), and BACS motor speed scores in FES patients. The correlation only between the ALFF of ROC-L and BACS motor speed scores, RSFC of STG-L and LG-L and BACS verbal memory scores survived for multiple comparisons (*P* < 0.007) (Figure 3).

## Discussion

We found a significant genotype × disease interactive effect of ALFF in the ROC-L, SMA-R (the brain area involved in this study is closer to its constituent structure, the supplementary eye field) and bilateral MOG in FES. Abnormal BOLD activities in these visual and auditory-related regional brain areas encouraged us to further detect whether dysconnectivity existed between these regions. Not surprisingly, a significant interactive effect was found of RSFC between the MOG-L and bilateral STG, STG-L, and several regions, including ROC-R, Cu-R, Fu-L, and LG-L, which are related to cognitive control (Kim et al., 2015). Increased BOLD activity and functional connectivity in schizophrenic patients may be a compensatory mechanism for the relatively inefficient activation of the related brain regions to achieve a near-normal level of neurocognitive performance even in the resting state (Kang et al., 2019). Moreover, patients with the TT genotype had higher ALFF values and RSFC strengths than patients with the A allele, which suggests possible effects on the activity of some brain regions by individual SAP97 rs3915512 variants.

In addition, the effects of SAP97 in schizophrenia may be limited to modulating the symptom profile; it may not confer risk for the disorder (Xu et al., 2018). Positive correlations were found between ALFF of ROC-L and motor speed scores, between RSFC of STG-L and LG-L and BACS verbal memory scores in FES. These results indicate that lower ALFF or RSFC have positive correlations with the severity of cognitive impairments in patients. Genetic variation and its possible contribution to brain activity and functional connectivity differences in FES may help explain individual differences in cognitive performance in patients with schizophrenia.

Higher SMA-R activity is likely leading to a more unsatisfactory performance on abstraction, oral comprehension, and short-term memory (Heiss et al., 1997). Located in the dorsal medial frontal lobe, the SMA receives direct nerve afferents from the thalamus and transmits efferent nerves to the striatum and dorsolateral prefrontal lobe, which are related to the generation and inhibition of motor activity, learning and cognitive control (Nachev et al., 2008). Using functional neuroimaging, increased functional connections between SMA-R and the bilateral cuneus in patients with schizophrenia were found in previous studies (Han et al., 2017). Complex auditory language tasks (repetition or conversion of sentences to passive forms) recorded the activation of ROC (Biermann-Ruben et al., 2005), indicating that ROC may play an essential role in the processing of auditory information. A cohort study of rehabilitation after stroke found extra activated ROC-L and gradually decreased activation degree of ROC-L with the recovery process (Van Dokkum et al., 2018). A positive correlation between ALFF of ROC-L and motor speed scores in this study was consistent with this finding, and a higher ALFF level in patients with the TT genotype may be more sensitive to therapy. MOG is involved in the encoding and extraction of visual information (Johnson et al., 2007). The increased activity of MOG has been consistently shown in schizophrenia (Galeno et al., 2004), which has a significant correlation with tests for verbal IQ, verbal learning, and executive functions (Hartberg et al., 2010).

Few reports mentioned the abnormal temporooccipital connectivity in schizophrenia before. The abnormal functional connectivity between the MOG-L and bilateral STG, STG-L and ROC-R, Cu-R, Fu-L, and LG-L has been mentioned in several cognitive disorders. The superior temporal lobe plays a role in language processing, attention, and integrative audiovisual functions (Makris et al., 2013). Its dysfunction in a range of cognitive tasks is a robust finding in functional neuroimaging studies of schizophrenia (Crossley et al., 2009). Friston and colleagues interpreted hyperactivation of the temporal lobe in schizophrenia during a verbal fluency task as a second-order effect of frontal lobe dysfunction (Friston et al., 2003). The main functions of the occipital lobe (including MOG, Cu, Fu, and LG) are processing visual signals, language and abstract thinking, and the abnormality in this area manifests as cognitive dysfunction, such as memory and abstract thinking, in patients with schizophrenia (Larabi et al., 2018). Our research results indicate that following previous studies, positive correlations were found between the RSFC of the STG-L and LG-L and BACS verbal memory scores, the most severe cognitive impairments in schizophrenia. In short, these visual/auditory-related pathways have been demonstrated to be directly or indirectly related to cognitive function in schizophrenia.

The allele frequencies of rs3915512 in our cohort from Zhanjiang (A:T = 0.24:0.76) were nearly similar to those of the population in southern China (A:T = 0.28:0.72), based on the 1000 Genomes Project.^2^ Drug-naïve FEP patients recruited for this study eliminated the possible interference of drugs. Several covariates and multiple correction reduced the rate of false positives. Therefore, our data may partly represent the potential effect of SAP97 in patients with schizophrenia in the Chinese Han population.

In summary, our results revealed abnormal BOLD activity and functional dysconnectivity in the auditory/visual-related brain areas in FES. Patients with the SAP97 risk allele appear to have more severe cognitive impairments and associated ALFF (except SMA-R) and RSFC of brain area reduction. Thus, the SAP97 rs3915512 polymorphism may affect cognitive function in schizophrenic patients by regulating brain activity and connectivity of auditory/visual-related brain areas.

## Data Availability Statement

The datasets generated for this study chave been added to dbSNP, Build (B156) (release Fall, 2020). The raw data supporting the conclusions of this article will be made available by the authors, without undue reservation.

## Ethics Statement

The studies involving human participants were reviewed and approved by the Ethics Committee of the Affiliated Hospital of Guangdong Medical University. Written informed consent to participate in this study was provided by the participants’ legal guardian/next of kin.

## Author Contributions

ZL, YW, and GM conceived and designed the experiments and revised the manuscript. XXW, JY, and XL did genetic analyzes. XW, SX, DZ, CL, and JF collected the clinical data. SL, DL, and ZD collected the imaging data. JL, YL, and WC analyzed and interpreted the data. XX, ZXL, and BH drafted the manuscript. All authors were involved in the revision of the manuscript.

## Conflict of Interest

The authors declare that the research was conducted in the absence of any commercial or financial relationships that could be construed as a potential conflict of interest.

## Footnotes

1 http://www.ali-velearn.net/xjview8/manual/

2 https://www.ncbi.nlm.nih.gov/variation/tools/1000genomes/

## Supplementary Material

Supplementary Table 1 22 MNI spatial coordinates of auditory and visual related brain areas.

Supplementary Table 2 Interactive effect and *post hoc* analysis of the ALFF value between the SAP97 rs3915512 genotype and disease.

Supplementary Table 3 Interactive effect and *post hoc* analysis of RSFC values between the SAP97 rs3915512 genotype and disease.
