# Supplementary material for: SAP97 rs3915512 Polymorphism Affects the Neurocognition of Schizophrenic Patients: A Genetic Neuroimaging Study
Source: Front Genet. 2020 Oct 8;11:572414. doi: 10.3389/fgene.2020.572414 (PMC7578398; doi:10.3389/fgene.2020.572414)
Supplement: Supplementary Table 1 — 22 MNI spatial coordinates of auditory and visual related brain areas. [file Table_1.DOC]

| Table S1. 22 MNI spatial coordinates of auditory and visual related brain area. | | | |
| --- | --- | --- | --- |
| AAL | MNI | AAL | MNI |
| left rolandic opercularis area | -38,-33,17 | left lingual gyrus | -15,-72,-8 |
| right rolandic opercularis area | 43,-23,20 | right lingual gyrus | 18,-47,-10 |
| left supramarginal gyrus | -50,-34,26 | left superior occipital gyrus | -14,-91,31 |
| right supramarginal gyrus | 59,-17,29 | right superior occipital gyrus | 15,-87,37 |
| left heschl gyrus | -55,-9,12 | left middle occipital gyrus | -24,-91,19 |
| right heschl gyrus | 45,-17,12 | right middle occipital gyrus | 40,-72,14 |
| left superior temporal gyrus | -49,-26,5 | left inferior occipital gyrus | -47,-76,-10 |
| right superior temporal gyrus | 58,-16,7 | right inferior occipital gyrus | 43,-78,-12 |
| left calcarine | -8,-81,7 | left fusiform gyrus | -33,-79,-13 |
| right calcarine | 8,-72,11 | right fusiform gyrus | 27,-59,-9 |
| left cuneus | -16,-77,34 |  |  |
| right cuneus | 15,-77,31 |  |  |
| MNI: Montreal Neurological Institute; AAL: Anatomical Automatic Labeling. | | | |
