# Supplementary material for: SAP97 rs3915512 Polymorphism Affects the Neurocognition of Schizophrenic Patients: A Genetic Neuroimaging Study
Source: Front Genet. 2020 Oct 8;11:572414. doi: 10.3389/fgene.2020.572414 (PMC7578398; doi:10.3389/fgene.2020.572414)
Supplement: Supplementary Table 2 — Interactive effect and post hoc analysis of the ALFF value between the SAP97 rs3915512 genotype and disease. [file Table_2.DOC]

| Table S2. Interactive effect and post hoc analysis of ALFF value between SAP97 rs3915512 genotype and disease. | | | | | | | | | | | |
| --- | --- | --- | --- | --- | --- | --- | --- | --- | --- | --- | --- |
| Brain region | |  |  | ALFF value | | | |  | | post hoc analysis of genotype in FES | |
| AAL | BA | MNI | Voxel (mm^3^) | HC | | FES | | Interactive effect | |  |  |
|  |  | X, Y, Z |  | TT | TA+AA | TT | TA+AA | *F* | *P* | *F* | *P* |
| SMA-R | 6 | 7, 0, 76 | 70 | 1.96±0.55 | 1.69±0.63 | 1.51±0.44 | 2.09±0.69 | 13.83 | 3.30E-04 | 13.16 | **4.52E-04** |
| ROC-L | 48 | -45, -30, 24 | 307 | 0.65±0.13 | 0.79±0.25 | 0.88±0.25 | 0.75±0.18 | 11.23 | 0.001 | 5.24 | 0.024 |
| MOG-R | 19 | 53, -75, 0 | 44 | 0.70±0.21 | 0.89±0.26 | 0.89±0.30 | 0.74±0.24 | 11.79 | 8.67E-04 | 4.53 | 0.036 |
| MOG-L | 19 | -48, -80, 16 | 58 | 0.74±0.15 | 0.89±0.25 | 0.86±0.22 | 0.70±0.17 | 15.70 | 1.40E-04 | 8.46 | **0.004** |
| AAL: Anatomical Automatic Labeling; BA: Brodmann area; MNI: Montreal Neurological Institute; HC: healthy control; FES: first episode schizophrenia; R: right; L: left; SMA: supplementary motor area; ROC: rolandic opercularis area; MOG middle occipital gyrus. | | | | | | | | | | | |
| Values are the mean± SD; 2× 2 ANCOVA *P*< 0.05 (Alphasim corrected, Cluster Size> 33); The bold values in the post hoc analysis can survive for Bonferroni correction (*P*< 0.0125). | | | | | | | | | | | |
|  |  |  |  |  |  |  |  |  |  |  |  |
|  | | | | | | | | | | | |
