# Supplementary material for: SAP97 rs3915512 Polymorphism Affects the Neurocognition of Schizophrenic Patients: A Genetic Neuroimaging Study
Source: Front Genet. 2020 Oct 8;11:572414. doi: 10.3389/fgene.2020.572414 (PMC7578398; doi:10.3389/fgene.2020.572414)
Supplement: Supplementary Table 3 — Interactive effect and post hoc analysis of RSFC values between the SAP97 rs3915512 genotype and disease. [file Table_3.DOC]

| Table S3. Interactive effect and post hoc analysis of RSFC values between SAP97 rs3915512 genotype and disease. | | | | | | | | | |
| --- | --- | --- | --- | --- | --- | --- | --- | --- | --- |
|  | | RSFC value | | | |  | | post hoc analysis of genotype in FES | |
| AAL | | HC | | FES | | Interactive effect | |
|  |  | TT | TA+AA | TT | TA+AA | *F* | *P* | *F* | *P* |
| STG-R | MOG-L | 0.29±0.23 | 0.41±0.21 | 0.35±0.22 | 0.19±0.26 | 11.59 | 9.64E-04 | 7.89 | **0.006** |
| STG-L | ROC-R | 0.45±0.22 | 0.67±0.20 | 0.61±0.32 | 0.47±0.28 | 14.24 | 2.77E-04 | 4.81 | 0.031 |
| STG-L | MOG-L | 0.25±0.19 | 0.42±0.18 | 0.36±0.24 | 0.18±0.27 | 16.69 | 9.08E-05 | 9.47 | **0.003** |
| STG-L | Cu-R | 0.21±0.25 | 0.38±0.17 | 0.37±0.22 | 0.25±0.24 | 12.43 | 6.46E-04 | 4.79 | 0.031 |
| STG-L | Fu-L | 0.31±0.15 | 0.44±0.22 | 0.37±0.26 | 0.22±0.25 | 11.32 | 0.001 | 7.15 | **0.009** |
| STG-L | LG-L | 0.30±0.20 | 0.41±0.18 | 0.40±0.21 | 0.24±0.28 | 10.25 | 1.85E-03 | 7.47 | **0.007** |
| AAL: Anatomical Automatic Labeling; HC: healthy control; FES: first episode schizophrenia; | | | | | | | | | |
| Values are the mean ± SD; 2 × 2 ANCOVA *P*< 0.002 ; The bold values in the post hoc analysis can survive for Bonferroni correction (*P*< 0.0125). | | | | | | | | | |
| R: right; L: left; STG: superior temporal gyrus; ROC: rolandic opercularis area; MOG: middle occipital gyrus; Cu: cuneus; Fu: fusiform, LG: lingual gyrus. | | | | | | | | | |
